# Single Nucleus RNA Sequencing Reveals Interaction Between Microglia and Endothelial Cells in Mixed Alzheimer’s Disease and Vascular Pathology

**DOI:** 10.1002/alz.089628

**Published:** 2025-01-03

**Authors:** Oluwatosin A Olayinka, Nicholas K O'Neill, Jenny A Empawi, Payton Bock, Junming Hu, Melissa Wong, Thor D. Stein, Benjamin Wolozin, Xiaoling Zhang, Lindsay A. Farrer

**Affiliations:** ^1^ Boston University, Boston, MA USA; ^2^ Boston University Chobanian & Avedisian School of Medicine, Boston, MA USA; ^3^ Boston University School of Medicine, Boston, MA USA; ^4^ Boston University Alzheimer’s Disease Research Center, Boston, MA USA; ^5^ Department of Pathology and Laboratory Medicine, Boston University Chobanian & Avedisian School of Medicine, Boston, MA USA; ^6^ VA Boston Healthcare System, Boston, MA USA; ^7^ Department of Veterans Affairs Medical Center, Bedford, MA USA; ^8^ VA Bedford Healthcare System, Bedford, MA USA; ^9^ Boston University Chronic Traumatic Encephalopathy Center, Boston University Chobanian & Avedisian School of Medicine, Boston, MA USA; ^10^ Boston University Chobanian and Avedisian School of Medicine, Boston, MA USA; ^11^ Department of Medicine (Biomedical Genetics), Boston University Chobanian & Avedisian School of Medicine, Boston, MA USA; ^12^ Boston University School of Public Health, Boston, MA USA; ^13^ Boston University Department of Medicine (Biomedical Genetics), School of Medicine, Boston, MA USA; ^14^ Boston University Bioinformatics Program, Boston, MA USA; ^15^ Department of Neurology, Boston University Chobanian & Avedisian School of Medicine, Boston, MA USA; ^16^ Boston University School of Medicine, Department of Medicine, Biomedical Genetics, Boston, MA USA; ^17^ Framingham Heart Study, Boston University Chobanian & Avedisian School of Medicine, Boston, MA USA; ^18^ Department of Epidemiology, Boston University School of Public Health, Boston, MA USA; ^19^ Departments of Medicine (Biomedical Genetics), Neurology, Ophthalmology, Biostatistics, and Epidemiology, Boston University Schools of Medicine and Public Health, Boston, MA USA; ^20^ Boston University Alzheimer’s Disease Research Center, Boston University Chobanian & Avedisian School of Medicine, Boston, MA USA; ^21^ Department of Biostatistics, Boston University School of Public Health, Boston, MA USA; ^22^ Alzheimer’s Disease Research Center, Boston University Chobanian & Avedisian School of Medicine, Boston, MA USA; ^23^ Department of Ophthalmology, Boston University Chobanian & Avedisian School of Medicine, Boston, MA USA

## Abstract

**Background:**

Single‐nucleus RNA sequencing (snRNAseq) allows for the dissection of the cell type‐specific transcriptional profiles of tissue specimens. In this study, we compared gene expression in multiple brain cell types in brain tissue from Alzheimer disease (AD) cases with no or other co‐existing pathologies including Lewy body disease (LBD) and vascular disease (VaD).

**Method:**

We evaluated differential gene expression measured from single nucleus RNA sequencing (snRNAseq) data generated from the hippocampus region tissue donated by 11 BU ADRC participants with neuropathologically confirmed AD with or without a co‐existing pathology (AD‐only = 3, AD+VaD = 6, AD+LBD = 2). Cell types were identified using both the singleR package and manual annotation. Expression of 19,893 genes was compared between samples with AD+VaD pathology and the other AD groups for each identified cell type. Gene co‐expression modules were identified using weighted gene co‐expression network analysis (WGCNA) applied to a partially overlapping group of 174 bulk RNAseq samples (AD‐only = 73, AD+VaD = 43, AD+LBD = 54, AD+other pathology = 4). Modules enriched in differentially expressed genes (DEGs) were identified using Fisher’s exact tests. The overlap between DEGs and co‐expression modules was used for quantitative gene set analysis. Replication of findings was performed on an independent cohort of 393 ROSMAP dorsolateral prefrontal cortex (DLPFC) snRNAseq samples.

**Result:**

Compared to other AD samples, AD+VaD subjects showed decreased expression of a specific set of genes related to immune activation in microglia (t = ‐2.67, p = 0.027). The expression these genes were negatively associated with that of microglial receptors *P2RY12* and *CX3CR1* (r = ‐0.87, p = 1.95 × 10^‐4^). We similarly observed a decrease in genes that negatively regulate angiogenesis in endothelial cells (t = ‐4.84, p = 1.48 × 10^‐3^). An association between the expression of the microglial activation and the endothelial anti‐angiogenesis gene set was found globally within the hippocampus snRNAseq data (r = 0.68, p = 1.59 × 10^‐2^) and was replicated in the ROSMAP DLPFC snRNAseq independent data set (r ‐0.60, p = 5.18 × 10^‐39^).

**Conclusion:**

We identified genes that are differentially expressed in endothelial cells and microglia in hippocampus from persons with AD+VaD compared to other AD diagnoses. These patterns suggest that AD+VaD cases have reduced microglial activation and increased angiogenesis in the brain.

